# Growing pains in children

**DOI:** 10.1186/1546-0096-5-5

**Published:** 2007-04-19

**Authors:** Yosef Uziel, Philip J Hashkes

**Affiliations:** 1 Department of Pediatrics, Pediatric Rheumatology, Meir Medical Center, Kfar-Saba, Tel-Aviv University, Israel; 2 Section of Pediatric Rheumatology, Dept. of Rheumatic Diseases, Cleveland Clinic Foundation, Cleveland OH, USA

## Abstract

We review the clinical manifestations of "growing pains", the most common form of episodic childhood musculoskeletal pain. Physicians should be careful to adhere to clear clinical criteria as described in this review before diagnosing a child with growing pain. We expand on current theories on possible causes of growing pains and describe the management of these pains and the generally good outcome in nearly all children.

## Background

A 6 year-old boy is awakened at 02:00 AM, screaming of painful legs. His parents appreciate severe pain, but do not see anything wrong with his legs. They rub his legs and give him acetaminophen and he returns to sleep with pain resolution within 30 minutes. In the morning he is running about as if nothing occurred at night. The following week he has a similar episode. This causes significant anxiety in the family, and they immediately visit the primary physician.

The family worries. What does he have? Is this a serious disease? A tumor?

What should the family physician do?

In this paper we will review the clinical presentation of this very common pain syndrome, try to understand current knowledge on the pathogenesis and natural course, what the family physician should do, especially in alleviating the family's anxiety.

Extremity pain is a common presenting complaint of visits to pediatricians [1-13]. The most common cause of childhood musculoskeletal pain is termed "growing pains" (GP) that exemplifies a type of non-inflammatory pain syndrome. These pains are much more common than all other inflammatory rheumatic diseases. The prevalence of GP ranges from 3–37% of children. Oster found that as many as 15% of school-age children have occasional limb pain [6], and recently Evans and Scutter in a very large community study in Australia reported a prevalence of 37% in children aged 4–6 years [7]. GP mainly affects children between the ages of 3–12 years.

GP has typical clinical characteristics; it is usually nonarticular, in 2/3 of children is located in the shins, calves, thighs or popliteal fossa and is almost always bilateral. The pain usually appears late in the day or is nocturnal, often awaking the child. The duration ranges from minutes to hours. The intensity can be mild or very severe. By morning the child is almost always pain free. There are no objective signs of inflammation on physical examination. GP is episodic, with pain-free intervals from days to months. In severe cases the pain can occur daily. In our series of 44 children with GP, 43% had frequency of at least once a week [14]. Often parents can predict when the child will have pain on days of increased activity or when the child is more moody.

GP is not associated with serious organic disease, and usually resolves by late childhood. However, frequent episodes may have a major impact on the child and his family's daily routine, including absences from school and work, daytime fatigue, reduced physical activity, and frequent or chronic use of pain relief medications.

## Pathogenesis

The etiology of GP is unknown. Very few studies have been done to elucidate the etiology and pathogenesis of this common syndrome.

## Low pain threshold

GP belong to the non-inflammatory musculoskeletal pain syndromes. Non-inflammatory pain syndromes, especially fibromyalgia, are associated with a lower pain threshold and with more characteristic tender points when measured by dolorimeter as compared to people without pain syndromes [15,16]. In fibromyalgia lower pain threshold and characteristic tender points are a dominant feature, which is found in the acute stage of the syndrome and resolves following remission [17].

Since GP is also a pain syndrome, our hypothesis was that the pain threshold in children with GP is lower compared to controls. We assessed the pain threshold by dolorimeter in 44 children with GP and indeed found that children with GP have a decreased pain threshold compared to age and gender matched controls [14]. It was interesting to note that the pain threshold at the anterior tibia was the highest in the body, including among children with GP. This is the region most commonly reported by children with GP as painful during attacks. This indicated that GP represents a generalized pain syndrome, not only a localized disorder. Thus it is possible that GP represents the pattern of non-inflammatory pain syndromes in early childhood.

## Bone strength

Since GP usually occurs late in the day and is often reported on days of increased activity, GP may represent a relative local overuse (stress) syndrome, and may be associated with decreased bone strength. In evaluating this theory we measured the bone speed of sound by ultrasound in 39 children with GP. We found that the bone strength density of children with GP was significantly less than values for population norms of healthy children, especially in the painful tibia region [18].

Thus GP may represent a local lower extremity overuse syndrome with bone fatigue in children with low pain thresholds. These children may experience more pain after physical activity. However while relative overuse can help explain late day pains, this theory cannot explain all features of GP such as the abrupt nocturnal episodes of pain, or pain in the upper extremity in some patients.

## Blood perfusion changes

The sudden onset and severity of GP as well as the transience of the attacks support a hypothesis that GP has a vascular perfusion component, similar to migraines. Furthermore a higher prevalence of GP was found among children with migraine headaches [19].

However when we looked for perfusion changes by comparing the ratio of blood phase of the bone scan to the static phase we did not find differences between children with GP and children who underwent bone scans for other reasons [20].

## Anatomical/Mechanical

Many clinicians have an impression that many children with GP are hypermobile, but this has not been assessed in a study in part due to the lack of formal criteria of hypermobility in very young children. This association, if true, may explain GP in 2 methods. GP after increased activity may be directly explained by hypermobility as part of the hypermobility syndrome. In addition children with hypermobility have an increased prevalence of fibromyalgia thus resulting in pain from a low pain threshold [21]. Other mechanical issues associated with hypermobility include flexible flat feet with hindfoot valgus. This mechanical instability might be a cause of GP in some children. In one small controlled trial shoe inserts were effective in reducing the frequency and severity of GP [22]. There is no evidence, however, that GP are actually associated with rapid growth as originally thought. The peak age of GP (about 6 years) is usually not part of the child's rapid growing phase.

## Family environment

Naish and Apley assumed that emotional disturbances are more common in children with GP, and that recurrent abdominal pain, headaches, and limb pains are a group of pain syndromes expressing a reactive pattern to familial emotional disturbances [4,23]. Oster suggested that painful experiences during the parents' childhood are a precipitating factor for development of a pain syndrome among their children [24]. In a study by Oberklaid et al, children with musculoskeletal pains (without the homogenous criteria of GP used for our studies) were often rated by their parents as having different temperamental and behavioral profiles than healthy normal controls, suggesting a psychosocial contribution to their pain, similar to that seen with other pain syndromes [25]. In others studies, the family environment and psychological distress were also found to contribute to the development of musculoskeletal pain syndromes [26,27].

We evaluated the quality of life, depression, and anxiety levels of parents to children with GP and found that the depression levels of the parents were similar to those from other non-inflammatory pain syndromes, with mothers having increased depression levels. Parents of children with GP and children without pain had similar quality of life scores, not surprising considering the episodic nature of GP [28].

## Other

Rarely, GP may be a manifestation of an organic disease like metabolic muscle disease (when occurring after exercise) or restless leg syndrome, especially in families with a history of this syndrome [29].

## Investigations and diagnosis

Currently, the diagnosis is based only on typical clinical symptoms as outlined above. There are no sensitive or specific laboratory tests, although children often undergo extensive investigations for other diseases. At least 19% of the children with GP undergo bone scans for evaluation of their pain [30]. When patients have typical clinical characteristics there is no need to do any laboratory or imaging tests. However, if the symptoms are atypical, the diagnosis of GP should not be assumed without evaluating other causes.

## Treatment

The most important intervention is to explain the natural benign course of the GP, thus decreasing anxiety and fear. Despite the benign prognosis, GP may have an impact on the child and family, especially among children with frequent nocturnal attacks. Comforting, local massage therapy during pain episodes or analgesics is used. Some children need to chronically use medications, especially acetaminophen and non-steroidal anti-inflammatory drugs (NSAID). In our study 52% of the children used medications to relieve their pain [14]. Occasionally nighttime use of a long acting analgesic, such as naproxen, may prevent episodes and can be used on days when parents predict an episode may occur or daily in children with frequent awakenings.

The calcium intake in our GP patients with lower bone strength group was relatively low [18]. It is possible that a diet enriched in calcium and vitamin D might affect bone status and pain episodes, but this theory was not investigated. We are not aware of any studies that have found an association between physical fitness and GP attacks.

Our findings of lower pain thresholds in children with GP may have therapeutic implications, such as behavioral intervention to decrease pain sensitivity (including cognitive behavioral therapy), as well as physical activity programs to increase fitness, which may decrease painful episodes. Physicians should be aware of the complete family psychological environment when treating children with GP and other non-inflammatory pain syndromes.

Other interventions shown to be effective in small controlled studies include in-shoe inserts such as triplane wedges or orthotics, especially in children with pronated foot posture [22], and a muscle stretching exercise program [31].

## Conclusions and future studies

GP is very common and easy to diagnose once the typical clinical characteristics are presented.

The natural history is benign with disappearance of most attacks of pain by adolescence. However it is not clear whether some of these children develop symptoms of other non-inflammatory pain syndromes. It would be important to progressively follow the pain thresholds of children with GP and to correlate the findings with their symptoms. Long-term outcome studies are suggested to investigate whether the children with GP who has a lower pain threshold, are prone to develop other non-inflammatory pain syndromes in the musculoskeletal or other systems later in adolescence or adulthood. If many children with GP do develop non-inflammatory pain syndromes later in adolescence or adulthood, a trial of early intervention, with cognitive behavioral therapy for example, may prevent progression to other syndromes later in life.

Further studies into the pathogenesis of GP should be performed. Further larger and genetically homogenous studies are required to determine whether the statistically significant findings of decreased bone SOS in our series of children with GP have clinical implication

## Abbreviations

GP: growing pains, NSAID: non-steroidal antiinflammatory drugs

## Competing interests

The author(s) declare that they have no competing interests.

## Authors' contributions

Both authors contributed to the collection of data and references, drafting and revising this manuscript. Both have read and approve the final manuscript.

## References

[B1] Passo MH (1982). Aches and limb pain. Pediatr Clin North Am.

[B2] Bowyer SL, Hollister JR (1984). Limb pain in childhood. Pediatr Clin North Am.

[B3] De Inocencio J (2004). Epidemiology of musculoskeletal pain in primary care. Arch Dis Child.

[B4] Naish JM, Apley J (1951). "Growing pains": a clinical study of non-arthritis limb pains in children. Arch Dis Child.

[B5] Brenning R (1960). Growing pain. Acta Soc Med Ups.

[B6] Oster J, Nielson A (1972). Growing pain: a clinical investigation of a school population. Acta Paediatr Scand.

[B7] Evans AM, Scutter SD (2004). Prevalence of "growing pains" in young children. J Pediatr.

[B8] Calabro JJ, Wachtel AE, Holgerson WB, Repice MM (1976). Growing pains: fact or fiction?. Postgrad Med.

[B9] Weiner SR (1983). Growing pains. Am Fam Physician.

[B10] Peterson H (1986). Growing pains. Pediatr Clin North Am.

[B11] Leung AKS, Robson WLM (1991). Growing pains. Can Fam Physician.

[B12] Manners P (1999). Are growing pains a myth?. Aust Fam Physician.

[B13] Atar D, Lehman WB, Grant AD (1991). Growing pains. Orthop Rev.

[B14] Hashkes P, Friedland O, Jaber L, Cohen A, Wolach B, Uziel Y (2004). Children with growing pains have decreased pain threshold. J Rheumatol.

[B15] Buskila D, Press J, Gedalia A, Klein M, Neuman L, Boehm R, Sukenik S (1993). Assessment of nonarticular tenderness and prevalence of fibromyalgia in children. J Rheumatol.

[B16] Yunus MB, Masi AT (1985). Juvenile primary fibromyalgia syndrome. A clinical study of thirty-three patients and matched normal controls. Arthritis Rheum.

[B17] Buskila D, Neuman L, Hershman E, Gedalia A, Press J, Sukenik S (1995). Fibromyalgia syndrome in children – an outcome study. J Rheumatol.

[B18] Friedland O, Hashkes PJ, Jaber L, Cohen A, Eliakim A, Wolach B, Uziel Y (2005). Decreased bone strength in children with growing pains as measured by quantitative ultrasound. J Rheumatol.

[B19] Aromaa M, Sillanpaa M, Rautava P, Helenius H (2000). Pain experience of children with headache and their families: a controlled study. Pediatrics.

[B20] Hashkes PJ, Gorenberg M, Oren V, Friedland O, Uziel Y (2005). Growing pains" in children are not associated with changes in vascular perfusion patterns in painful regions. Clin Rheumatol.

[B21] Gedalia A, Press J, Klein M, Buskila D (1993). Joint hypermobility and fibromyalgia in schoolchildren. Ann Rheum Dis.

[B22] Evans AM (2003). Relationship between growing pains and foot posture in children: single case experimental designs in clinical practice. J Am Podiatr Med Assoc.

[B23] Apley J (1970). Clinical canutes: A philosophy of pediatrics. Proc Royal Soc Med.

[B24] Oster J (1972). Recurrent abdominal pain, headache and limb pain in children and adolescents. Pediatrics.

[B25] Oberklaid F, Amos D, Liu C, Jarman F, Sanson A, Prior M (1997). "Growing Pains": clinical and behavioral correlates in a community sample. J Dev Behav Pediatr.

[B26] Sherry DD (2000). An overview of amplified musculoskeletal pain syndromes. J Rheumatol.

[B27] Palermo TM (2000). Impact of recurrent and chronic pain on child and family daily functioning: a critical review of the literature. J Dev Behav Pediatr.

[B28] Uziel Y, Friedland O, Jaber L, Press J, Buskila D, Wolach B, Hashkes PJ Living with children with growing pains: How does it affect the parents?. J Musculoskel Pain.

[B29] Rajaram SS, Walters AS, England SJ, Mehta D, Nizam F (2004). Some children with growing pain may actually have restless leg syndrome. Sleep.

[B30] Macarthur C, Wright JG, Srivastava R, Rosser W, Feldman W (1996). Variability in physicians' reported ordering and perceived reassurance value of diagnostic tests in children with growing pains. Arch Pediatr Adolesc Med.

[B31] Baxter MP, Dulberg C (1988). "Growing pains" in childhood: a proposal for treatment. J Pediatr Orthop.

